# Digitalization of toxicology: improving preclinical to clinical translation

**DOI:** 10.3389/ftox.2024.1377542

**Published:** 2024-03-28

**Authors:** Brian R. Berridge, Szczepan W. Baran, Vivek Kumar, Natalie Bratcher-Petersen, Michael Ellis, Chang-Ning Liu, Timothy L. Robertson

**Affiliations:** ^1^ B2 Pathology Solutions LLC, Cary, NC, United States; ^2^ VeriSIM Life, San Francisco, CA, United States; ^3^ The Jackson Laboratory, Bar Harbor, ME, United States; ^4^ TLR Ventures, Redwood City, CA, United States; ^5^ Drug Safety Research and Development, Pfizer, Groton, CT, United States

**Keywords:** toxicology, artificial intelligence, machine learning, digital, biomarker

## Abstract

Though the portfolio of medicines that are extending and improving the lives of patients continues to grow, drug discovery and development remains a challenging business on its best day. Safety liabilities are a significant contributor to development attrition where the costliest liabilities to both drug developers and patients emerge in late development or post-marketing. Animal studies are an important and influential contributor to the current drug discovery and development paradigm intending to provide evidence that a novel drug candidate can be used safely and effectively in human volunteers and patients. However, translational gaps—such as toxicity in patients not predicted by animal studies—have prompted efforts to improve their effectiveness, especially in safety assessment. More holistic monitoring and “digitalization” of animal studies has the potential to enrich study outcomes leading to datasets that are more computationally accessible, translationally relevant, replicable, and technically efficient. Continuous monitoring of animal behavior and physiology enables longitudinal assessment of drug effects, detection of effects during the animal’s sleep and wake cycles and the opportunity to detect health or welfare events earlier. Automated measures can also mitigate human biases and reduce subjectivity. Reinventing a conservative, standardized, and traditional paradigm like drug safety assessment requires the collaboration and contributions of a broad and multi-disciplinary stakeholder group. In this perspective, we review the current state of the field and discuss opportunities to improve current approaches by more fully leveraging the power of sensor technologies, artificial intelligence (AI), and animal behavior in a home cage environment.

## Introduction

Our use of animals as surrogates for humans to better understand human health and disease has presented a significant and growing challenge. Despite the historical value and impact of animal studies, there are concerns that the data generated from animals is not as translationally relevant as we have believed it to be and often not replicable (van der Worp et al., 2010; Kimmelman and Federico, 2017). Additionally, animal studies are increasingly viewed as unethical and that novel, non-animal modeling systems using human cells (e.g., microphysiological systems [MPS]) can provide improved insights and perform better than traditional animal models (Low et al., 2020).

Animal studies have been a foundation for basic biomedical research and advanced our understanding of human biology and disease for those pathophysiologies that are conserved between animal model species and humans. The use of animals in research has also supported a drug discovery and development enterprise that has produced a growing portfolio of effective and safe drugs, vaccines, and medical devices that extend and improve the quality of life of patients, both human and animal. Throughout the drug development process, animal studies are influential in validating novel drug targets, modeling drug metabolism and disposition, demonstrating the pharmacological action of a drug candidate, and informing our ability to safely progress that drug candidate to human clinical trials. Accordingly, we have justified our need to study animals because of their unique ability to model human-relevant complexity and integration of biology as no other existing modeling platform can do.

Safety assessment is a key component of the drug development process that includes both preclinical animal studies and human clinical trials. Preclinical approaches to drug safety assessment are highly animal-dependent and often involve a series of standardized animal studies in two species (rodent and non-rodent) of varying duration, design, and intent. This portfolio of studies includes general toxicity studies, developmental and reproductive studies, immunotoxicity assessments, and functional assessments of important organ systems like the cardiovascular, central nervous, and respiratory systems. The primary aims of drug safety assessment studies are to identify potential human toxicities, characterize those toxicities (e.g., morphology, dose-relatedness, reversibility, etc.) and inform whether they can be effectively monitored and managed in human clinical trials. The endpoints we collect in our animal safety studies are generally those that we collect when evaluating health and disease in human patients including serum biochemistry, hematology, urinalysis, assessments of organ function, and histopathology. Though self-reported and observed behaviors are fundamental to the clinical assessment of human patients and provide important insights into systemic health and disease, preclinical assessments of animal behavior are mostly relegated to specific applications and rarely evaluated quantitatively-e.g., the Functional Observational Battery (FOB) CNS safety pharmacology studies (Redfern et al., 2019). Further, spontaneous behavior is not a routine biological endpoint in most studies other than subjective cage-side clinical observations as a general health check. A routine physical examination in a patient would generally include questions to the patient related to their historical sense of wellbeing, activity levels, eating and drinking, pain, and other indicators of potential systemic disease. The inability of an animal to communicate those perspectives necessitates inferring them from more manual and episodic examination of natural or evoked behaviors like posture, gait, grip strength, tremors, startle response, and hair coat quality among others. Though those assessments are relevant to human patients and translatable, they are collected discontinuously, during the light period when rodents are usually sleeping, and in the presence of an observer which naturally alters the animal’s responses.

Complementing our use of animal studies is a rapidly growing portfolio of novel platforms and assays that enable us to generate biological data at unprecedented volumes and levels of biological and mechanistic resolution (e.g., at the molecular level). The recently passed FDA Modernization Act 2.0 promotes a greater acceptance of non-traditional data spurring evermore novel approaches (Anonymous, 2023). Our ability to generate rapidly expanding volumes of data has catalyzed the development of computational capabilities that can reasonably manage, integrate and leverage that data-i.e. an age of digitalization. That digitalization has yet to substantively extend to animal studies.

## Problem statement

Drug discovery and development is a challenging business. It takes a long time (frequent estimates of 10–15 years), fails often (<10% of candidates that enter human clinical trials are eventually marketed), and costs a lot (estimates of $2.5 billion/market-approved medicine (May et al., 2023). Safety assessment studies and the liabilities identified (or not) in those studies are important contributors to those challenges. Animal safety studies are costly, time-consuming, and low throughput. Safety liabilities identified in preclinical animal studies can lead to late-stage termination just prior to the onset of clinical trials where safety assessments are generally performed in development. Worse yet, safety liabilities that emerge in human clinical trials that were not predicted by preclinical animal studies (i.e., translational failures) put patients at risk. Significant portions of the data collected from preclinical animal studies like histopathology assessments and clinical observations are subjective (even when generated by expert observers), often not quantitative, and difficult to integrate with data generated in other parts of the drug development process undermining their replicability.

As noted above and by the Food and Drug Administration (FDA) Center for Drug Evaluation and Research (CDER), traditional approaches to drug safety assessment which include both *in vivo* and *in vitro* assessments have generally performed well in protecting patients from unintended harm (Avila et al., 2020). This is particularly true for safety liabilities that manifest in changes in usual endpoints like histopathology, clinical pathology analytes, and acute functional studies. Alternatively, drug-induced neurobehavioral effects like dizziness, nausea, and somnolence are common in patients, often lead to patient intolerance, but are not predicted well by animal studies using traditional endpoints (Cook et al., 2014). These translational gaps have contributed to concerns that animal studies are leading us astray. A number of these predictive challenges were recently represented by Avila et al. (2023), which included several neurobehavioral gaps in our current approaches including the need for more standardization of safety pharmacology protocols, more quantitative and objective measures in core neurological screens, and improved prediction of human-relevant seizure risk. These translational challenges have several possible contributors. Innate biological differences between animal species and strains used in safety assessment studies and human patients can reasonably lead to different outcomes. Conducting studies in two or more animal species as expected by regulatory agencies is an attempt to mitigate those comparative biological differences but that undermines our intent to minimize the number of animals used in research (Ledwith and DeGeorge, 2011). As noted above, the data collected from animal studies is often subjective and susceptible to unintended bias. Additionally, most of the endpoints are collected at the termination of a study and are not continuous or dynamic in the way biology and pathobiology actually happens. Accordingly, the pathogenesis (i.e., the onset, duration, progression, and recovery) of a pathobiological change is often left to speculation (albeit by pathologists who are trained to do that speculating). Compounding that challenge is recognizing that most of the behavioral data routinely collected in animals is cursory and collected during the day, which is convenient for researchers, but disruptive for animals like rodents who are nocturnal and sleep during the light cycle.

Despite the enactment of the FDA Modernization Act 2.0, animal safety studies remain a necessary and important contribution to our efforts to develop safe and effective medicines, but they would benefit from a better use of rapidly developing technologies that could make them more efficient, translational, replicable, amenable to integration with other data streams, and even fewer in number (Anonymous, 2023).

## Opportunity statement

The way we conduct animal drug safety studies has not kept pace with rapid advances in technology, data science, and our growing understanding of biology and pathobiology. Better and bolder use of rapidly developing technological capabilities could significantly improve the replicability and human translational predictivity of animal studies.

More holistic monitoring and digitalization of animal studies have the potential to enrich study outcomes leading to datasets that are more computationally accessible, translationally relevant, replicable, and technically efficient. Digitalization of drug development overall has been evolving for a number of years but experienced a significant increase in pace during the recent pandemic, prompting ways of working that require less direct human involvement (May, 2023).

Digitalization currently exists at a modest level in safety assessment as safety pharmacology studies where continuous and quantitative measures of major organ function (e.g., heart rate, electrocardiogram, blood pressure, body temperature, etc.) are collected from animals that are implanted with sensors (i.e., telemetry, radio frequency identification [RFID] microchips). Also, digitalization is being leveraged in pathology where histologic sections of tissues on glass slides are being digitized to facilitate sharing, computational evaluations, and artificial intelligence (AI)-assisted image analysis (Ge et al., 2022). Digitization of animal behavior and non-invasive monitoring of important physiological functions like respiratory rate and locomotion could represent the next level of animal study digitalization and provide a stronger link to the human clinical setting where behavior is already a usual measure of health and wearable sensors are providing more continuous measures of physiology (Peng et al., 2019; Winn et al., 2021).

**FIGURE 1:**
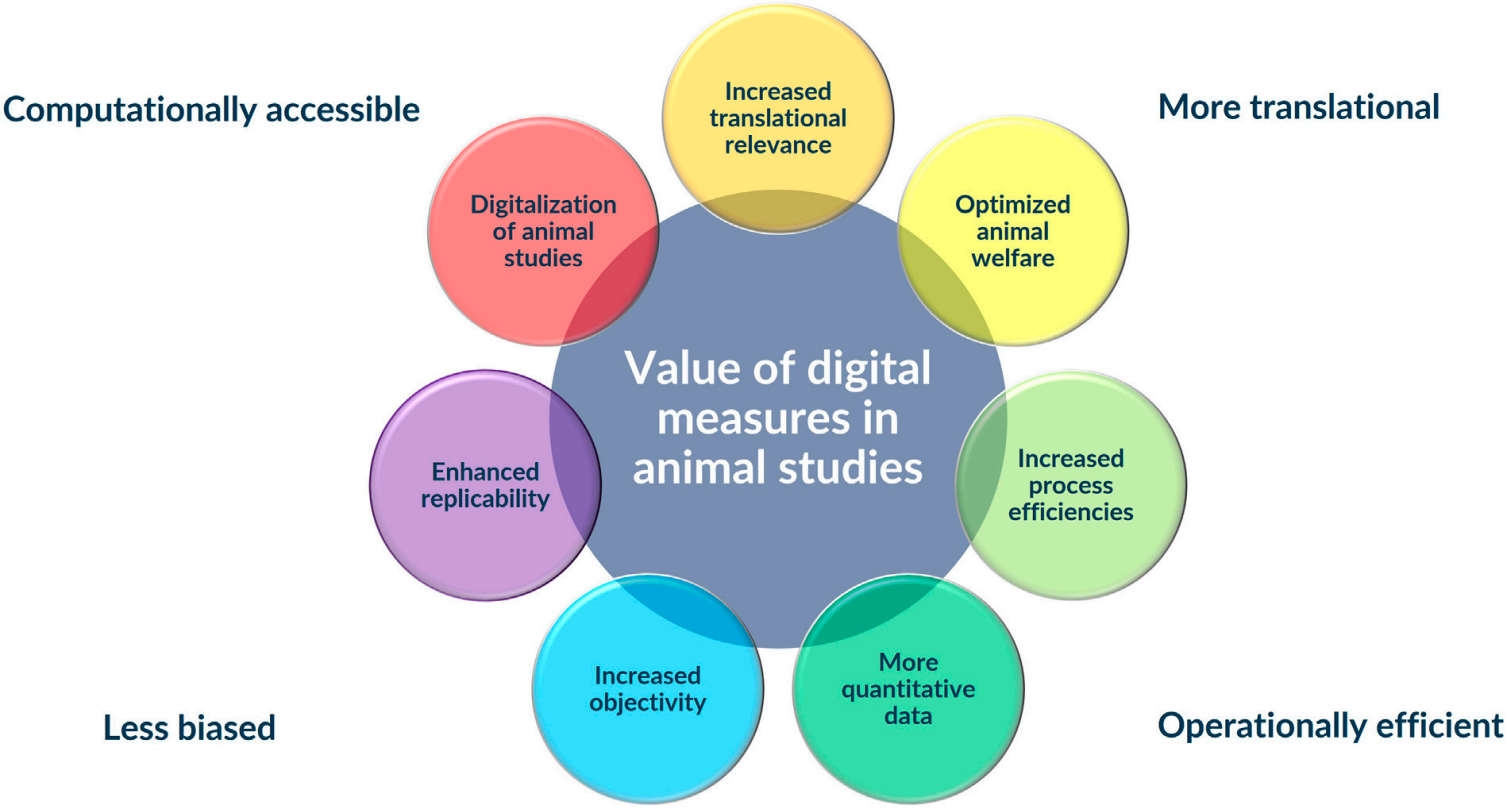
Integration of digital measures into traditional animal safety assessments studies has the potential to add substantial value to animal studies improving their human translation, making them more computationally accessible, less biased, and more operationally efficient.

Complementing our usual biochemical, hematological, and histopathological assessments with continuous measures of behavior and physiology collected in the home cage would provide a more dynamic, integrated, and clinically relevant characterization of potential drug safety liabilities (Figure 1). Continuous monitoring during both the light and dark cycles would support higher fidelity correlations between drug effects and the toxicokinetics of the parent drug or its metabolites. Identifying behavioral correlates to systemic toxicities could provide less invasive biomarkers of those toxicities enhancing monitoring and mitigation strategies. Continuous measures would also allow earlier detection of animal welfare concerns (e.g., impending mortality) and the potential for re-defining “maximum tolerated dose” thresholds that could be more humane. Automated measures would mitigate human biases and reduce subjectivity, improving the replicability of animal studies. Digitally quantitative measures can be better integrated across the drug development evidence streams, allowing better use of AI- and machine learning (ML)-based approaches that not only make the collection of usual endpoints more efficient, but also provide access to novel endpoints to provide unique insights into the beneficial and adverse bioactivities of our drug candidates in the preclinical phase of testing. More specifically, identifying behavioral correlates to some of the drug-induced neurological effects that are currently not predicted by usual endpoints but continue to vex patients (e.g., seizure, dizziness, somnolence, nausea) would be a major advance in our predictive capabilities (Authier et al., 2013). More holistic, dynamic, and integrated assessment of both the pharmacological and non-pharmacological effects of new medicines would enable a better opportunity to manage the balance between benefit and risk inherent in any novel therapeutic.

## Digital solutions

Usual safety assessment studies include both continuous and discontinuous measures of important biological endpoints though the final data set is heavily biased toward discontinuous data. As noted above, surgically implanted and telemetered sensors are used commonly in rodent and non-rodent safety pharmacology studies to provide sensitive and continuous measures of critical physiological functions like heart rate, electrocardiogram, blood pressure, respiration, body temperature, etc. (Ewart et al., 2013). Those measures are most often collected in single or short duration dose studies to identify acute life-threatening effects in target organ function prior to initiation of human clinical trials.

Routine assessments of animal behavior are most often conducted using cage-side observations. These observations can reveal obvious test article-related effects but are also performed as a routine health check to ensure animal welfare. More structured and sensitive assessments of behavior are included in the FOB that is the basis for a CNS Safety Pharmacology assessment. The FOB is usually conducted during the light cycle and outside the animal’s home cage (e.g., open field) representing an exceedingly “unnatural” assessment (Redfern et al., 2019).

**FIGURE 2:**
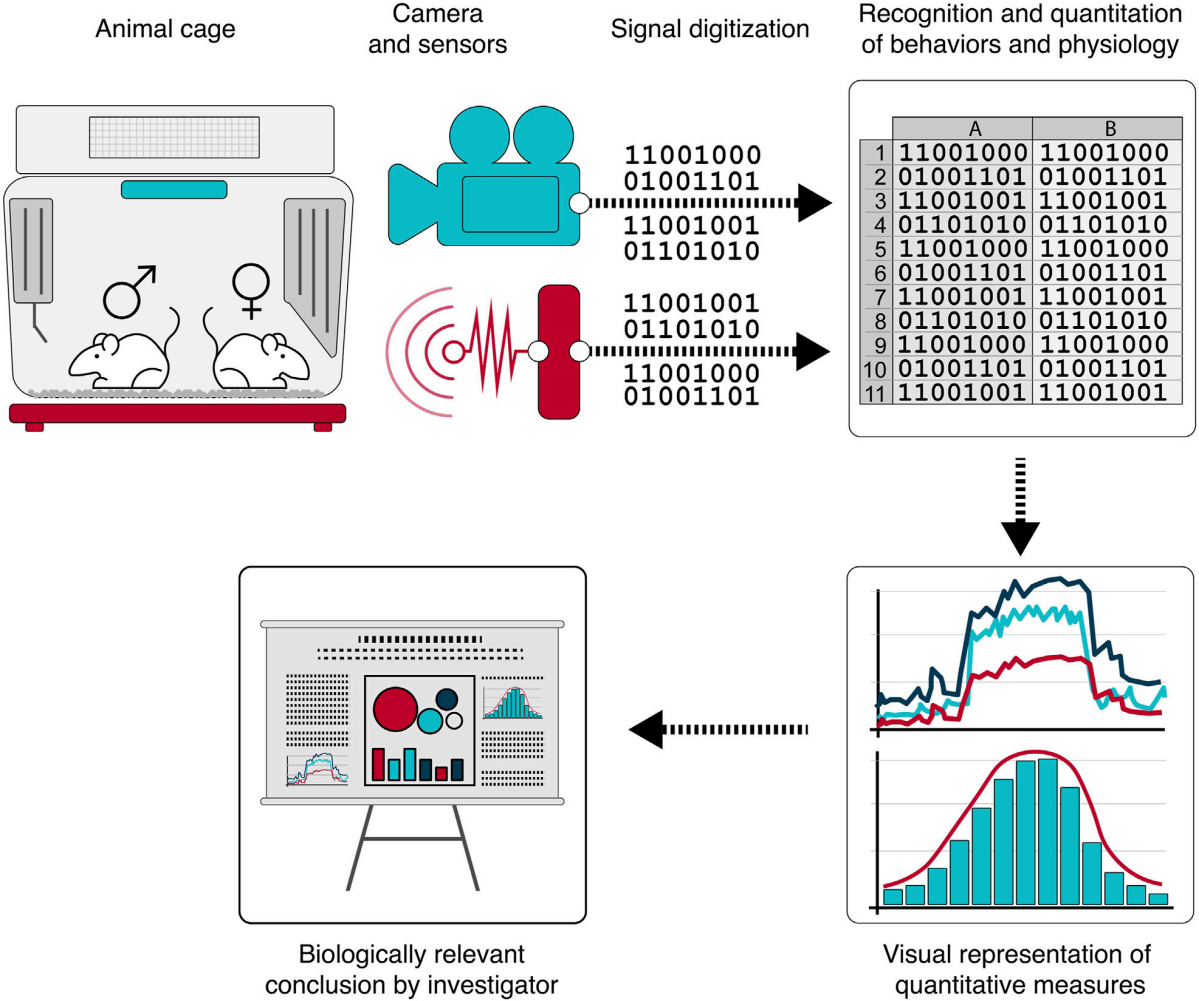
A growing portfolio of sensor technologies are being applied to and continuously monitoring rodent home cage behavior. Application of machine learning algorithms to the digitized monitoring data produces computationally and analytically accessible, quantitative, and objective data supporting clinically relevant interpretations and decision-making.

There are several emerging home cage-based technologies using various approaches like electrical impedance, photovoltaic beams, RFID, and computer vision to quantitatively and continuously monitor breathing, body temperature, locomotion, or activity in rodents (Baran et al., 2021). Continuous monitoring of group-housed rodents in a home cage environment using these sensors, complemented by ML-derived computational algorithms offers the opportunity for a revolutionary “digitalization” of animal studies—including animal safety studies (Figure 2). Well-defined and validated “digital measures” are substrate for broader digital integration across the drug development process, offering an opportunity to more fully leverage “behavior” as a relevant and translational physiological endpoint with greater objectivity in measures of response or effect. Behavioral endpoints could provide important insights into the onset and progression of organ system disease allowing for a more dynamic characterization of pathogenesis and its relationship to internal exposure kinetics.

Digitalization, in its simplest form, involves using automated approaches to collect analog, continuous, quantitative, biological, and environmental data that can be computationally digitized, organized, analyzed, integrated, and reported. Collecting the data in a home cage environment with less disruption to the animal subjects provides logistical efficiencies, but also less artifact. Real time analysis of digital data enables more adaptive study designs, increasing the speed of decision-making, decreasing the need for repeat-studies, and alerting the experimenter to impending animal health/welfare issues. The archival nature of digital data also supports continuous learning from individual studies as capabilities for detecting and quantifying specific behaviors increase and understanding of the behavioral correlates to systemic disease improves. Also, digitalized *in vivo* experiments become the substrate for *in silico* simulation and predictive modeling approaches that will progressively enhance our preclinical assessments and complement our animal studies by better informing their design and interpretation. Data from digitalized animal studies could be used along with historical *in vivo* study data for building virtual control groups, which could partly or entirely replace concurrent controls (Golden et al., 2023). A broader use of objective and quantitative digital measures would also support a more analytical rules-based approach to decision making that would be more defensible, engender greater confidence, and allow for more managed risk-taking. The outcomes of those decisions become the substrate for an iterative training of the “rules” progressively improving the quality of the decisions. In a drug development context, this improvement could reasonably translate to reduced attrition and more frequent clinical successes.

There is precedent for demonstrating the unique value of these novel digital approaches. Ho et al. (2023) developed a home cage video-based tracking system that was able to identify changes in memory, learning, locomotion and rest or quiescence in animals with chemically-induced hippocampal and entorhinal lesions. Gschwind et al. (2023) demonstrated better performance in differentiating epileptic from non-epileptic mice during their interictal period using a video-based motion sequencing approach relative to human observers. Likewise, the approach was able to distinguish behavioral progression of the experimentally induced epilepsy as well as anti-epileptic drug response. Tse et al. (2018) demonstrated the unique ability to identify a delayed decrease in activity in rats given chlorpromazine using a video and RFID-based monitoring system relative to a traditional modified Irwin assessment. Also, Zhang et al. (2022) detected decreases in average paw luminance ratio using an infrared camera sensor under a cage bottom in freely moving mice modeling pain induced by a spectrum of experimental stimuli.

Lastly, digitalization of animal studies would facilitate integration with other sources of data in the drug development repertoire of assessments including those where quantitative data is more usual (e.g., *in vitro* studies) allowing better alignment of biological mechanisms or modes of action and phenotypic outcomes.

## A roadmap

Adopting novel and innovative ways of working is not without difficulty. Despite broad recognition of the need to continuously improve our current approaches, novel approaches bring uncertainty of acceptance by relevant stakeholders (including regulators) and the likelihood of significant impact on drug development success. Experience and outcomes build confidence in value. Accordingly, it is likely that the adoption of digital measures will evolve with experience.

Though behavioral assessments of animals are common in basic neuroscience research and are applied routinely in drug safety assessment with some understanding and confidence in the translational relevance of the underlying biology, most of those assessments are done episodically, during light cycles, are contrived, and in artificial environments undermining their translational predictivity. More routine and continuous application of behavioral monitoring leveraging recent and rapid developments in sensor technology and AI-based algorithm development will provide a better understanding of animal behaviors, how they reflect systemic disease, and how they relate to analogous biology in human patients. We are on the front end of an innovative approach to the way that we do animal studies more fully leveraging behavior as an important physiological endpoint that will add value in the near term and increase that value as we gain more knowledge and confidence to expand its use.

Continuous monitoring and digital measures can be readily applied in the exploratory pre-IND phase of safety assessment potentially supporting candidate selection and providing opportunity for early identification of liabilities. Confidence and experience could lead to initially complementing, and ultimately replacing specific assessments like the CNS FOB. Therapeutic targets or drug classes with known neuroactivity would be reasonable triggers for applying a more enhanced and continuous assessment of behavior. The greatest value will come when we have broadened our portfolio of validated digital measures and applied them routinely in general safety and toxicity assessments to complement standard endpoints where they have the potential to significantly improve the translational predictivity and replicability of preclinical studies.

The regulatory landscape for acceptance of digital drug development approaches including sensor technology, AI-defined algorithms, and digital biomarkers or health measures is rapidly evolving and probably most mature for clinical applications. The FDA’s Digital Health Center of Excellence (https://www.fda.gov/medical-devices/digital-health-center-excellence) is a useful source of information and guidance for clinical approaches. For preclinical approaches, the FDA Center for Drug Evaluation and Research’s (CDER) Drug Development Tools Qualification Program (https://www.fda.gov/drugs/development-approval-process-drugs/drug-development-tool-ddt-qualification-programs) and Innovative Science and Technology Approaches for New Drugs (ISTAND) Pilot Program (https://www.fda.gov/drugs/drug-development-tool-ddt-qualification-programs/innovative-science-and-technology-approaches-new-drugs-istand-pilot-program) provide useful guidance for engaging the Agency and building their confidence in these novel sources of data. Likewise, medicines regulatory authorities in Europe are also developing their experience with digital measures in healthcare and drug development (Colloud et al., 2023).

## Discussion

Concerns about the value and human-relevance of animal studies are not well supported by the benefits that have come from our history of using them. Animal studies have significantly advanced our understanding of human and animal biology and health and have protected most patients from unintended harm by novel therapeutics. However, there are enough examples of outcomes in animals that were not reproduced in human patients to prompt efforts to improve the human relevance of animal studies. Also, the biomedical research community has an obligation to use animal studies judiciously which includes ensuring that they are contributing to an effective and sustainable model of drug discovery and development.

Drug safety assessment is a standardized and structured enterprise designed for consistency and to enable key decisions that inform the clinical progression of novel drug candidates. The current portfolio of studies that are conducted meet usual regulatory expectations defined in agency guidance. The evolution of that portfolio over the 80+ years it has been codified in policy and regulation has been prompted by gaps that emerged and our growing understanding of how drug-induced toxicity manifests in animals and human patients. Substantively changing this historical approach is not without challenge.

Rapid and ongoing advances in sensor and computational technologies are an opportunity to improve the translational and logistical efficiencies of animal studies used in drug development- including in safety assessment. A digitalization of animal studies has the potential to improve their analytical power, translational relevance, and impactful “shelf life.” Digital measures derived from continuous computer vision monitoring or other sensor technologies and defined by ML-based algorithms can support many of the benefits to be gained from that digitalization. More routine, continuous, sensitive, and specific assessments of animal behavior as important physiological endpoints akin to usual clinical practices have the potential to improve the translational relevance of animal studies. It also has the potential to provide a more complete understanding and characterization of the pathogenesis of disease-including toxicity.

A digital revolution in the way animal studies are conducted will best come from dynamic and multi-disciplinary collaborations. Safety assessment and animal scientists articulate the questions and the decision context for those questions. Engineers design and build sensors that collect biologically meaningful data. Data scientists build the capabilities to manage and analyze that data applying the rapidly developing capabilities of AI and ML. Regulators represent the risk assessment context by managing the cage to clinic translation of the data we collect to ensure patient benefit and safety.

Several multi-disciplinary collaborations and resources have recently developed to support the adoption and application of digital measures in both preclinical and clinical drug development. The Digital Medicine Society (dimesociety.org) supports the development and use of digital medicine approaches to enhance patient care. The 3Rs Collaborative (na3rsc.org) initiates and manages efforts to advance the 3Rs (refine, reduce, replace) of animal research including an aim to support the use of translational digital biomarkers. COST (European Cooperation in Science and Technology) TeaTime (cost-teatime.org) brings together European organizations developing and using automated home-cage monitoring technologies. Most recently, the Digital *In Vivo* Alliance (DIVA; diva.bio), a group of biopharmaceutical company scientists, veterinarians, and data scientists, is working collaboratively to advance digital measures for preclinical *in vivo* research through their development, validation, adoption, and regulatory acceptance. Each of these efforts is contributing to a growing body of evidence supporting the value of digital measures in preclinical animal studies. As experience and the community of stakeholders grow, standards and best practices will emerge. Collectively, these efforts have great potential for supporting a timely and effective development and application of digital measures in animal studies which should substantially contribute to their effective support of progressing effective and safe medicines to patients. The pace and magnitude of that contribution depends on the level of effort committed by the stakeholders.

## Data Availability

The original contributions presented in the study are included in the article/supplementary material, further inquiries can be directed to the corresponding author.
